# Assessment of the Occurrence of Forest Fires in Pandemic Period by COVID-19 in Chile. Preliminary Backgrounds

**DOI:** 10.3390/ijerph181910529

**Published:** 2021-10-07

**Authors:** Miguel Castillo, Jorge Saavedra, Tomás Quiñones, Tatiana Osses, María José Torres

**Affiliations:** 1 Forest Fire Laboratory, University of Chile, Santiago 9206, Chile; 2 National Forestry Corporation, Santiago 285, Chile; jorge.saavedra@conaf.cl (J.S.); tomas.quinones@conaf.cl (T.Q.); tatiana.osses@conaf.cl (T.O.); maria.torres@conaf.cl (M.J.T.)

**Keywords:** wildfires, COVID-19, quarantine, wildfire risk

## Abstract

The spatial and temporal behavior of the occurrence of forest fires in Chile was evaluated in the presence of COVID-19 and mobility restrictions. The fire period from 2015–2016 to 2020–2021 was considered and statistics on mobility restrictions were granted by the Government of Chile. The analysis was developed at different scales of geographic perception. At the national and regional levels, the global behavior of the occurrence was determined, and later at the communal level, the political territorial unit, to determine internal variations attributable to the mobility dynamics in the quarantine period. In the process, the meteorological background of the fire activity was also considered. The results indicate that it is possible to rule out a meteorological effect, based on the variation of the moisture content of fine fuel. There was also no statistical association between the humidity of the fuel and the variation in the occurrence of fires. It is concluded that the communes that presented the greatest mobility of people before the pandemic were those that obtained the greatest reduction in fires. The variation in mobility, the product of restriction measures, is a statistical predictor of the increase or decrease in fires.

## 1. Introduction

The COVID-19 pandemic has threatened millions of human lives and devastated social and economic conditions globally [1]. On 11 March 2020, COVID-19 was declared a global pandemic by the World Health Organization. A year and three months later, on 29 June 2021, 181 million cases of COVID-19 had been registered worldwide, including more than 4.4 million deaths [2]. The effects have even been transferred to the environment, including the interaction between confinement and air quality dependent on various environmental activities and events, including forest fires. In this last context, the impact of forest fires on human health has been widely studied from the point of view of gas emissions resulting from the combustion of vegetation. In addition, the effect of smoke has been related to respiratory difficulties caused by the COVID-19 virus in periods that coincide with the confinement of the population and the occurrence of fires [3], as well as from the point of view of virus exposure and air pollution in highly vulnerable communities from a socio-economic and cultural point of view [4].

The effect of confinement and COVID-19 levels has also been studied in 34 countries using mobility data reported in Google and Apple [5]. In this study, they determined that the levels of pollution and exposure to COVID-19 decrease significantly when applying confinement measures. However, little is known about the existence of possible relationships between confinement and fires. A study carried out in Colombia [6] concluded that the restriction measures affecting the mobility of the population and its confinement as protection measures against contagion by COVID-19 have a direct impact on the number of fires and their extension. This same study indicates that the increase in the number of fires was also produced by the lack of control and surveillance in areas prone to activities that produce them. Another study developed in Nepal [7] indicates that COVID-19 caused a decrease of 4.54% in the number of forest fires and a reduction of 11.36% in radiative power generated by the fire associated with these events. The estimates from this study also showed that districts with smaller areas of forest managed by local communities experienced an 8.11% decrease in the number of fires.

However, these references are not conclusive for all regions of the world, mainly because each country approaches differently the policies to protect its population against the pandemic, and because the climatic seasonality and origin of forest fires are aspects. that frequently cannot be compared between one country or another, even between regions of the same country or municipality [8]. In Sub-Saharan Africa, the effect of confinement and mobility restrictions caused by COVID-19 on air quality and carbon dioxide emissions was studied [9]. It was found that there was an increase in emissions, attributable to less control in the areas of fire occurrence, and with it more extensive events in terms of propagation. However, they did not find conclusive evidence regarding containment measures and a reduction in the number of fires. 

In a context further away from forest fires, and more associated with socioeconomic and sociocultural aspects linked to the presence of the pandemic, [10] mapped the racial and economic inequalities in access to health due to COVID-19, indicating clear weaknesses in the local management of the pandemic. In Chile, the “Paso a Paso” (“Step by Step”) plan of the Ministry of Health [11] establishes the restrictions on people and communities and activities in the context of the pandemic. Its implementation made it possible to provide visibility and transparency to citizens regarding what it means to move forward or backward and what each step implies. This plan has been adjusted in accordance with the recommendations of experts and feedback from the affected people and communities, in order to reconcile a plan that better balances the health objectives and the social effects produced by the restrictions contained in the COVID-19 response plan. Part of the data generated from the application of this step-by-step plan, in particular the quarantine periods, allow for the establishment of mobility patterns that could be related to activities associated with disturbances, including forest fires.

In Chile, the occurrence statistics have not changed substantially in the last 15 years. Indeed, the fluctuations from one year to the next are part of the statistical behavior, with some years slightly away from the normal mean. The reduction in the number of fires has been attributed to other factors, including the climate component, and in 2020 and 2021, to the reduction in the mobility of people as a result of the health restrictions applied by the advance of the COVID-19 pandemic, and the macro-climatic phenomenon of La Niña, which cyclically affects the coasts of the Pacific Ocean. However, these latter causes that could explain a decrease in the occurrence are not properly documented with statistical evidence, an aspect that this research wishes to address precisely. Regarding the expansion of fires, before the COVID-19 pandemic, a constant increase in the number of large-scale fires was reported, which in the case of this country are classified as such when they exceed 200 hectares affected by the fire. The hazard condition has also been on the rise, due to the cumulative effects of drought, which have been expressed in the scarcity of rainfall and increased water stress in the forests, regardless of whether they accommodate native trees or exotic plantations.

As for the causes of the fires that occur in Chile, they have been concentrated in very specific origins: intentional and irresponsible use of fire. This trend has been maintained in the last 40 years, with slight variations. The meteorological factor plays a fundamental role in understanding the conflict of forest fires. Although it is true that the probability of occurrence tends to be associated with episodes of high temperatures, winds, and low relative humidity, as well as to the moisture content of fine vegetation, it is common to find extreme weather conditions not necessarily associated with the increase in the occurrence of forest fires. For this reason, fire risk and danger are associated, but do not form a cause–effect relationship [8]. For this research, the hypothesis is that the state measures to reduce mobility gradually applied between regions of the country affected by the COVID-19 pandemic, and also at the level of communes in a more detailed analysis, influenced the decrease in the number of forest fires detected for the period 2020–2021.

## 2. Materials and Methods

### 2.1. Study Area

**Figure 1:**
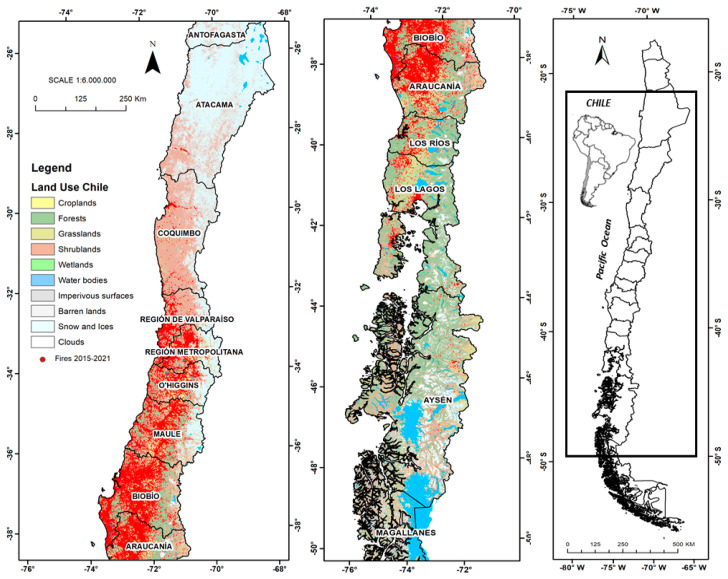
National diagnosis of the occurrence of wildfires in Chile. Period 2015–2021. Areas highlighted in red indicate the distribution and concentration of forest fires in the south-central region of Chile.

The research was carried out between the Atacama and Magallanes regions, fully covering the geographic dispersion of forest fires in Chile. The central-south zone concentrates the largest number of fire events (Figure 1), and where a warm temperate climate is present with a marked seasonality, typical of a Mediterranean climate, comprising high temperatures in summer and low rainfall, coinciding during the period of fires with a low humidity of fine and dry vegetable fuels, which are the main promoter of combustion when the ignition factor is present. Prolonged drought and climate change have contributed to the growing presence of more extreme events and greater conflict regarding control over the means of extinction. In Chile, most fires occur between the months of December and February, with gradual variations between months of the year. Figure 1 shows a general aspect of the spatial distribution of fires at the level of the main regions of Chile (scale 1:1,500,000), for the period 2015–2021. In essence, the density distribution pattern is concentrated in the proximity of the main cities and roads, a situation that is closely related to the mobility and activity of people, because in Chile, the main cause of fires is attributed to the presence of humans. In terms of figures, during the 2016–2017 season, when huge fires occurred that affected more than 600,000 hectares of forests and scrub, the number of fires did not vary significantly.

### 2.2. Wildfire Occurrence

At the regional level, the figures for the occurrence of forest fires vary, depending on the internal characteristics of each territory. In this research, a preliminary study of the figures was carried out, considering the 2015–2020 period (five fire seasons) as an analysis window, to make interannual comparisons for the period from 15 March 2020 to 15 March 2021, coinciding with the evolution of the pandemic in Chile due to COVID-19. To develop the hypothesis, regarding the effect of the mobility reduction measures on the number of fires registered for the period 2020–2021, an analysis of standardized anomalies was carried out, in order to determine variations in the number of forest fires for each region and commune.

(1) $$\mathrm{Anomaly}=\frac{{\bar{X}}_{\begin{array}{c} occurrence\\ 2020-2021 \end{array}}\_{\bar{X}}_{\begin{array}{c} History\;\\ occurrence \end{array}}}{{\;\sigma }_{\mathrm{History}\;\mathrm{occurrence}}}$$

The historical occurrence is the mean and standard deviation of the periods 15 March (current year)–15 March (next year) for the seasons 2015–2016, 2016–2017, 2017–2018, 2018–2019, and 2019–2020. This was undertaken for both perception scales, regional and communal. So, this is an indicator of variation of fire occurrence for the current period, that is, under mobility restrictions.

For the analysis at the communal scale, the communes of all Chile were selected whose historical mean was higher than the average at the level of all the communes of Chile. Due to the internal variability of the behavior of forest fires at the commune level (local scale), a data collection was defined incorporating preferably statistical criteria. For this selection, those communes that behave as anomalous data (outliers) were identified, specifically those that exceed the upper limit of the occurrence values. The identification of these outlier data was performed using the method described by McGill et al. (1978) [12], whereby data of this type are defined in quartiles, with those that exceed the third quartile of the total data distribution being processed. This process was carried out using the R language and the databases of the statistical system of the National Forestry Corporation.

### 2.3. Meteorology

The statistical link between occurrence under a period of mobility restriction and the meteorological variable was studied. In the case of forest fires, the most relevant variable corresponds to the humidity condition of fine and dead vegetation [13]. For its evaluation, the dew point and surface temperature were analyzed, because they are variables that directly affect variation in the humidity of the vegetation. Original data for this process were obtained from the ERA-5 (satellite sensor) data collection from Copernicus Climate Data Store [14]. For this, the adjusted model of relative humidity RH of Alduchov and Eskrigde (1996) [15], expressed in Equation (2), was applied. Images were selected for the pandemic and historical period and processed using Google Earth Engine code. Specifically, the indicator of conditions conducive to the propagation of the fire chosen is the humidity of the fine and dead fuel at 3:00 p.m., when the radiation is maximal, and the humidity tends to be the minimum of the day. The result of this was the determination of the minimum average moisture value of the fine and dead fuel, for the entire area and period of investigation. After that, different time ranges were established to evaluate current and historical periods. Furthermore, to calculate anomalies, it was necessary to estimate the deviation of the historical data as well. All these steps are indicated in the script and the remaining scripts were written with the purpose of exporting the data and later analyzing them in programming language. Subsequently, this result allowed to determine the moisture of the fine and dead fuel (FFMC) by means of the expression indicated in (3), and that corresponds to the forest fire expansion simulator of the KITRAL System developed at the University of Chile and statistically validated with real fires [8]. Indeed, this simulator was tested and successively in field studies of fire behavior under different environmental conditions [13], and permanently revised and used in various forest fires that occurred in the study area until the year 2021.

RH = 100 × (exp((17.625 × TD)/(243.04 + TD))/exp((17.625 × T)/(243.04 + T)))(2)

FFMC (%) = −2.97374 + (00262 × RH) − (0.00982 × T)(3)

Estimates of FFMC anomalies were made for the current period on a regional scale in order to identify potential climatic scenarios that could have an effect on the occurrence. The relationship between this variation of FFMC min and the variation of fire occurrence, both expressed as anomalies, was also evaluated. A Pearson coefficient of determination was estimated to evaluate the relationship between both variables.

### 2.4. Mobility

**Figure 2:**
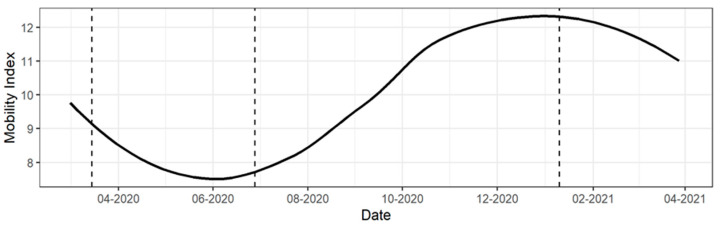
Sample of a mobility process applied to the Valparaíso Commune in Central Chile. 3 defined periods, from left to right: 1. Pre-Quarantine Mobility, 2. Quarantine Mobility, 3. Post-Quarantine Mobility.

Information regarding the mobility of people was obtained from a recent research in Chile [15] in which daily mobility indices were estimated at the communal level, from the period prior to mobility restrictions to the present. This index, generated from telecommunications antennas, describes relative values of internal and external mobility of people in a commune. Then, 3 events associated with the communal mobility time series were defined. According to this index [16], pre-quarantine mobility is described as the mean values of the mobility index for the period between 9 March and 15 March 2020, before the establishment of confinement measures in Chile. Quarantine mobility is described as the average for the period between 22 June and 28 June 2020, the one with the greatest restriction to national mobility. Additionally, a third event was defined, namely post-quarantine mobility, between 3 January and 9 January 2021, at which time the Chilean government began to allow holiday transfers between regions [17], which began to increase mobility, as described in Figure 2.

To calculate an explanatory model, 3 variables were reformulated that describe the mobility dynamics at the community level for the period to be evaluated.

[Mob1]: Pre-Quarantine Mobility

[Mob2]: Pre-Quarantine Mobility/Quarantine Mobility

[Mob3]: Pre-Quarantine Mobility/Post-Quarantine Mobility

Mob1 is an indicator of the normal mobility of the commune without restrictions, whereby the greater the magnitude, the greater the mobility. Mob2 is an indicator of the variation produced by mobility restrictions, whereby values higher than 1 indicate a decrease in mobility as a result of these restrictions. Mob3 is an indicator of the restoration of mobility. When mobility reached maximum levels with respect to the evaluated period, values greater than 1 indicate a restoration less than the conditions prior to mobility restrictions.

## 3. Results

### 3.1. Wildfire Occurrence

The results that mark the greatest differences are shown in Table 1. At the national level, the greatest reductions in the occurrence in the pandemic period are concentrated in the central zone of Chile, particularly in the regions of Coquimbo and Valparaíso. The statistical behavior of the remaining regions can be attributed to other factors, which are not necessarily explained by the mobility restrictions in the pandemic.

In accordance with the above and on a national scale, it is possible to affirm that the current season, under mobility restrictions, is described as having a lower occurrence of fires, with a negative anomalous value exceeding the historical deviation of the data.

On a regional scale, those that exceeded at least one standard deviation of the historical occurrence are Coquimbo, Valparaíso, and los Ríos, indicating a significant decrease in terms of occurrence. On the other hand, only Aysén had a significant increase in occurrence.

**Figure 3:**
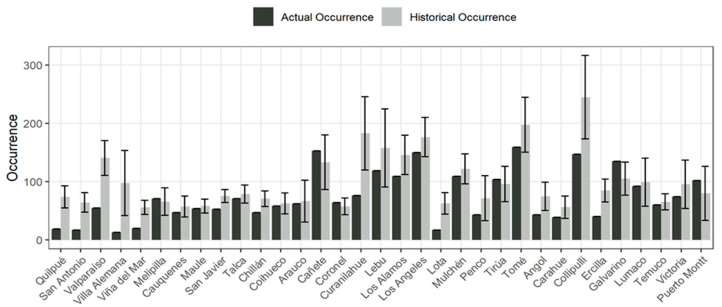
Occurrence Lockdown and historical. Communal scale. Period 15 March 2020–15 March 2021.

At the community level, the national median of occurrence in Chile is 9.4 fires for the period (15 March 2020–15 March 2021). The interquartile range (25–75%) is [3.225, 22.6]. According to the outlier detection method [12], communes were selected whose historical mean exceeds 51.6 fires for the period covered. The result described 33 communes out of a total of 325 (Figure 3), within the study area (Atacama to Magallanes). It is necessary to indicate that this selection is statistical, so it does not consider the local problem of fires, which can be very different from one commune to another. In this sense, this research corresponds to a first reference that can later be studied in greater detail by analyzing the local situation of each commune with more data and variables.

According to Figure 3, and considering the anomaly calculation method described in Equation (1), the communes that present a more ostensible decrease in the number of fires during a pandemic belong to the central region of Valparaíso, specifically in the communes of Quilpué (lockdown occurrence with 19 fires, historical 74 ± 19 and anomaly of −2.92), San Antonio (lockdown occurrence with 17 fires, historical 64 ± 17 and anomaly of −2.79), Valparaíso (lockdown occurrence with 55 fires, historical 141 ± 30 and anomaly of −2.87, and Viña del Mar (lockdown occurrence with 20 fires, historical 56 ± 12 and anomaly of −2.93. Other communes located further south of Chile also presented strong downward anomalies of fires, such as Lota and Curanilahue (−2.45 and −1.7 respectively) in the Bio Bío Region, as well as Ercilla and Collipulli (−2.28 and −1.37 respectively) in the Araucanía Region.

### 3.2. Meteorology

The hypothesis raised concerning the effects of reduced mobility on the number of fires was complemented with the analysis of large-scale meteorological data. The incidence of local meteorology was not included since the available data correspond to a geographical scale that does not allow to provide reliable data on the dependence of this variable on the effect on fire reduction and mobility. The processed data of the ERA-5 data collection for the capture of the dew point and air temperature allowed to determine the humidity of the fine and dead fuel (FFMC). Moreover, 20 years of data were processed to determine a climatic temporal pattern and compare it with the minimum data obtained for the time series of 15 March 2020–15 March 2021. The results obtained for all the regions of the study area show that there is no significant variation between the historical mean of minimum values and the observed mean for the pandemic period (Table 2).

**Figure 4:**
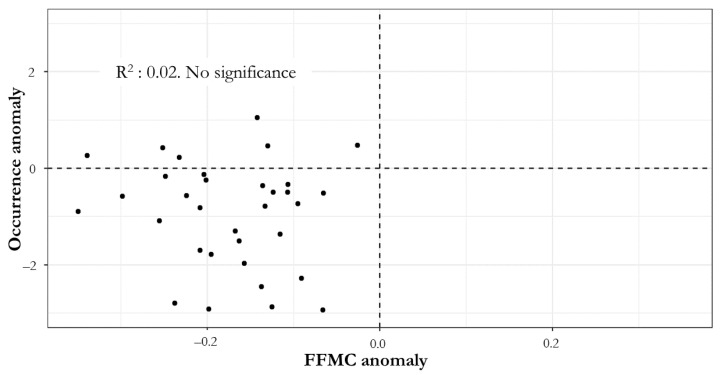
Relationship Between FFMC Anomalies and Occurrence at the Communal Level (Selected Communes).

The Fine Fuel Moisture (FFMC) represents fuel moisture of forest litter fuels under the shade of a forest canopy. As indicated in Table 2, there are no significant anomalies in any of the regions, as all of them are within the historical deviation of the data. Regarding the relationship between the variation in the occurrence of forest fires and fuel humidity for the 2020–2021 pandemic period, the scatter graph in Figure 4 shows that there is no trend that represents a clear measure of association between these two variables, at the community level and for the pandemic period analyzed.

### 3.3. Relations between the Occurrence of Forest Fires and People Mobility

**Figure 5:**
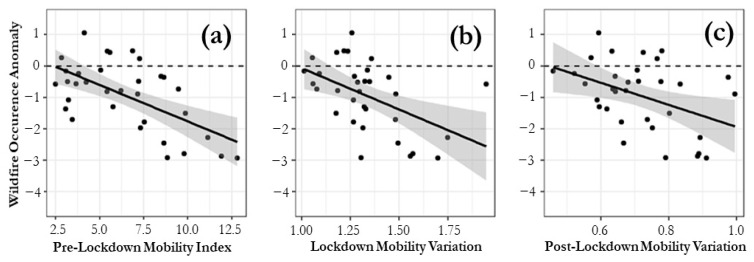
Linear adjustment of the fire occurrence anomaly for the selected communes and each of the mobility variables: Mob1, Mob2 and Mob3.

A multiple linear regression was calculated to predict forest fire occurrence anomalies based on the community mobility parameters: Pre-Quarantine Mobility (Mob1), Quarantine-associated Variation (Mob2), and Post-Quarantine Variation (Mob3). A significant regression was found (F (3,29) = 9.303, *p* < 0.05) with an R^2^ of 0.49. It could be predicted that the occurrence anomaly is 2.27−0.24 (Mob1) − 2.65 (Mob2) + 2.61 (Mob3). Both Pre-Quarantine Mobility (a) and Quarantine-Associated Variation (b and c) were significant predictors of anomalies in fire occurrence (Figure 5). The standard error of this model is 0.83.

A statistical relationship was identified between the dynamics of mobility of people and the variation in the occurrence of fires. In the same way, it was found that those communes characterized by high mobility prior to the restrictions were those that had a significant decrease in fires. When considering the variation of mobility, as a result of restriction measures, there was a better explanation for the increase or decrease in occurrence. Then, those territorial units with high mobility prior to the restrictions, and where the application of these measures had a strong impact on it, were those with the greatest decrease in fires.

## 4. Discussion

The mobility figures at the regional level and later at the communal level show very marked comparative differences between the months of June 2020 and January 2021, mainly due to the measures applied for mobility and quarantine restrictions (Table 3). In this regard, detailed information is available on the confinement phases, defined in the national “Step by Step” plan of the Government of Chile [18], as well as in the instructions for displacement [17]. In effect, the application of measures contained in these government instructions allowed for the elaboration of a detailed information base regarding the mobility of people, which added to the references on indices based on analysis of cellular telephone signals [16] for modeling the variations in the mobility of people, which consequently made it possible to have information for the purposes of this re-search associated with the occurrence of forest fires and the study of anomalies attributable to the presence of the COVID-19 pandemic. In a context more associated with the behavior of the population in areas where a wide range of activities coexist in addition to the presence of fires, the importance of developing statistical models that allow studying in greater detail other activities associated with the internal mobility of each commune arises, as well as where the determination coefficients R^2^ are normally low and the statistical significance of the relation of occurrence versus confinement and mobility is usually scarce. Indeed, the results of Figure 3 also express positive anomalies in the occurrence of fires in the confinement period.

These positive anomalies are part of the seasonal fluctuation of the occurrence of fires, which provides insufficient evidence to determine an inverse relationship between confinement and the increase in the number of fires. In this research, the density of fires per unit area was not considered, because the comparison period corresponds to only one year of data (period of onset and development of the pandemic in Chile), while the number of fires per unit of territorial data is normally determined with periods of at least 5–10 years of accumulated data.

Regarding the meteorological variable described for the research area, the humidity condition of the fine and dead vegetation remained at medium levels, not determining a direct association with the decrease in the occurrence of fires at the regional and communal levels. The aforementioned bibliographic references associate the presence of forest fires in the confinement period, from the point of view of fire behavior, the average size of the burned areas, and the emission of greenhouse effect gases.

## 5. Conclusions

There are statistical associations between anomalies derived from the variation in the occurrence of fires and the phases of mobility according to a review at the communal level. Indeed, those communes that reported greater mobility prior to the confinement measures are those that subsequently reduced the rate of fires the most during the application of restrictions. However, in the selection of the 33 communes for this research, it was not possible to consider other antecedents associated with the details of the occurrence and local causes of fires, which could make a commune much more important in the analysis, and which does not appear in the 33 selected here.

The model developed here is a first reference to understand the association between the occurrence of forest fires and confinement in a pandemic period. In order to obtain better results, especially at the commune level, it is necessary to incorporate variables of a social, economic, and behavioral order with respect to the usual activities carried out by the population when it is subjected to special protection measures and limited displacement. Indeed, at the regional level, the relationship between these variables was more robust than in the more detailed analysis, mainly because at the local level other aspects related to the behaviors and activities of people that are not necessarily associated with the risk of starting fires are involved.

An interesting piece of information to investigate in a more detailed analysis of the occurrence of fires is their causes, as they could be associated with more specific behaviors of the population that carry out activities in areas with a high risk of forest fires.

Consequently, the research hypothesis is partially true, since the relationship between the occurrence variable and its anomalies is fulfilled for a regional analysis, while at the commune level, it is partially explained by the mobility phases applied according to the “Step by Step” plan of the Government of Chile. Indeed, this hypothesis is not fulfilled in six of the 33 selected communes, a relationship that is attributable to other socio-economic factors that are not possible to study in detail based on the data used here.

An important aspect to be considered for a more detailed study corresponds to the accumulated distribution of the occurrence of fires as the season progresses in each region or commune, because the general average for a particular period does not allow to obtain the activity dynamics in respect of fire events within a territorial unit.

Regarding the meteorological variable, the cyclical presence of the La Niña phenomenon on the coasts of the Pacific Ocean determined a negative anomaly in practically all the regions of the research area, but with an insufficient magnitude to conclude statistical dependence between anomalies in the occurrence of fires and the condition of fine and dead vegetation, based on the calculation of dew point and air temperature.

This research will make it possible to contribute to the knowledge base in terms of monitoring the occurrence behaviors when protection measures and mobility controls are implemented in the fire risk variable, which in the case of Chile is preferably associated with human activity.

## Figures and Tables

**Table 1:** Current wildfire occurrence anomaly (15 May 2020–15 May 2021), under mobility restriction, and historical.

Region	Occurrence (Lockdown)	Occurrence (Historical)	Anomaly
Atacama	18	23 ± 6	−0.83
Coquimbo	58	96 ± 10	−3.80
Valparaíso	432	864 ± 107	−4.03
Metropolitana	363	420 ± 93	−0.61
O’Higgins	239	295 ± 74	−0.76
Maule	801	847 ± 172	−0.27
Ñuble	425	494 ± 54	−1.27
Biobío	1.719	2.032 ± 441	−0.71
Araucanía	1.080	1.195 ± 334	−0.34
Los Ríos	82	120 ± 35	−1.08
Los Lagos	269	188 ± 84	0.96
Aysén	48	30 ± 13	1.38
Magallanes	19	18 ± 9	0.11
National	5553	6622 ± 903	−1.18

**Table 2:** FFMC anomaly (%) for the current season. Estimated historical values within the range 2001–2020 (20 years).

Region	Lockdown FFMC	Historic FFMC	Anomaly
Atacama	3.2	3.6 ± 1.8	−0.26
Coquimbo	6.7	7.4 ± 1.6	−0.42
Valparaíso	8	8.8 ± 2	−0.40
Metropolitana	8.1	8.8 ± 2.8	−0.26
O’Higgins	9.1	9.7 ± 3	−0.19
Maule	9.7	10.2 ± 3.3	−0.15
Ñuble	10.1	10.7 ± 3.3	−0.19
Biobío	11	11.5 ± 3.1	−0.16
Araucanía	12.7	13 ± 2.8	−0.13
Los Ríos	13.9	14.2 ± 2.5	−0.11
Los Lagos	15	15.3 ± 2.5	−0.12
Aysén	16.5	16.7 ± 2.2	−0.09
Magallanes	17.5	17.3 ± 1.7	0.11

FFMC = Fine Fuel Moisture.

**Table 3:** Communes with positive anomalies in the occurrence of forest fires. Period 15 May 2020–15 May 2021.

Region	Commune	Lockdown Occurrence	Historic Occurrence	Anomaly
Metropolitana	Melipilla	71	66 ± 24	0.23
Bio Bío	Cañete	153	133 ± 47	0.43
	Coronel	64	57 ± 14	0.47
	Tirúa	104	96 ± 30	0.26
Araucanía	Galvarino	135	105 ± 28	1.05
Los Lagos	Puerto Montt	102	80 ± 46	0.48
